# Influence of Fluorinated Polyurethane Binder on the Agglomeration Behaviors of Aluminized Propellants

**DOI:** 10.3390/polym14061124

**Published:** 2022-03-11

**Authors:** Chen Shen, Shi Yan, Yapeng Ou, Qingjie Jiao

**Affiliations:** State Key Laboratory of Explosion of Science and Technology, Beijing Institute of Technology, Beijing 100081, China; 3120185140@bit.edu.cn (C.S.); ouyapeng@bit.edu.cn (Y.O.)

**Keywords:** fluorinated polyurethane, thermal decomposition, agglomeration, aluminized propellants, combustion performance

## Abstract

In this study, fluorinated polyurethane (FPU) was prepared from dialcohol-terminated perfluoropolyether as a soft segment; isophorone diisocyanate (IPDI) as a curing agent; 1,2,4-butanetriol (BT) as a crosslinker; and 1,4-butanediol (BDO) as a chain extender. Fourier transform infrared spectroscopy (FTIR) and ^1^H NMR were used to characterize the structure of the FPU. The mechanical properties of the FPUs with different BDO and BT contents were also measured. The tensile strength and breaking elongation of the optimized FPU formula were 3.7 MPa and 412%, respectively. To find out the action mechanism of FPU on Al, FPU/Al was prepared by adding Al directly to FPU. The thermal decomposition of the FPU and FPU/Al was studied and compared by simultaneous differential scanning calorimetry-thermogravimetry-mass spectrometry (DSC-TG-MS). It was found that FPU can enhance the oxidation of Al by altering the oxide-shell properties. The combustion performance of the FPU propellant, compared with the corresponding hydroxyl-terminated polyether (HTPE)-based polyurethane (HPU) propellant, was recorded by a high-speed video camera. The FPU propellants were found to produce smaller agglomerates due to the generation of AlF_3_ in the combustion process. These findings show that FPU may be a useful binder for tuning the agglomeration and reducing two-phase flow losses of aluminized propellants.

## 1. Introduction

Polyurethane binder is an important component of composite solid propellants, which function as a matrix to provide dimensional stability for filler particles [1,2]. The evolution of composite solid propellant energy performance is usually accompanied by the innovation of the binder system. As the binder evolved from the initially developed bituminous to hydroxyl-terminated polybutadiene (HTPB) and hydroxyl-terminated polyether (HTPE), the specific impulse of the propellants increased gradually from 176 to 270 s [3,4]. Although these new composite solid propellants have superior energy performance, the agglomeration of aluminum combustion, usually existed in these high-aluminized HTPB and HTPE propellants, often brings a series of nonignorable problems [5,6,7]. The agglomeration of Al prolongs the combustion interface between aluminum and oxygen, resulting in incomplete thermal energy conversion and high two-phase flow losses [8,9]. It has been reported that for every 10% of unburned Al, the specific impulse (*I*_sp_) loss is approximately 1% [10]. Moreover, the agglomeration of Al also causes adverse effects on the working process of the motor system, such as slag accumulation, nozzle erosion, and unsteady motion inside the combustion chamber [11,12].

To avoid problems associated with the agglomeration of Al, recent efforts have focused on the addition of fluorine-containing polymers to Al powders [13,14,15]. The oxidation reaction of Al-fluorine has a higher energy density than that of Al-oxygen [14]. More importantly, fluorine-containing polymers such as polytetrafluoroethylene (PTFE), polyvinylidene difluoride (PVDF) and homemade organic fluoride have proven to be effective in reducing agglomeration [16,17,18]. Wen et al. [16] prepared fluoropolymer-coated Al composite particles, which showed a shorter ignition delay time and smaller agglomerates during propellant combustion. Guo et al. [17] found that adding organic fluorides can significantly decrease the particle size and residual active aluminum in the condensed combustion products of aluminized propellants. However, most of the reported fluoropolymers are used as additives rather than binders in solid propellants because of the solid-state of common fluoropolymers or the limitations of the preparation process [16,19]. Therefore, finding a suitable FPU binder is expected to reduce the agglomeration of Al of aluminized propellant and avoid the above drawback.

In this paper, FPU was successfully prepared via the curing reaction of a liquid fluorine-containing prepolymer. The FPU was subsequently used as a binder in the aluminized propellant for the purpose of improving agglomeration. FTIR, ^1^H NMR and mechanical tests were used to analyze the structural and mechanical properties of FPU. DSC-TG-MS, SEM, EDS and high-speed photography were used to analyze the role of FPU loading with respect to decomposition behavior and combustion performance. In addition, a mechanism for the suppression of agglomerate size in the combustion of FPU/AP/Al propellants is postulated.

## 2. Materials and Methods

### 2.1. Materials

All the materials utilized in the experiments were of analytical grade. Dialcohol terminated perfluoropolyether (Fluorolink, E10-H) was purchased from Solvay Company (Brussels, Belgium), with an average equivalent weight (NMR) of 750. Hydroxyl-terminated polyether (HTPE) was obtained from Liming Research Institute of Chemical Industry (Luoyang, China), with an average molecular weight of 2800 and hydroxyl value of 0.73 mmol/g. Isophorone diisocyanate (IPDI), triphenylbismuth (TPB), 1,2,4-butanetriol (BT) and 1,4-butanediol (BDO) were purchased from Aladdin Biochemical Technology Co., Ltd. (Shanghai, China) E10-H, HTPE, BT and BDO were dried under vacuum at 70 °C before use to ensure the removal of moisture. Ammonium perchlorate (AP, 220 μm and 20 μm) was provided by Jiangyang Chemical Industry Co., Ltd. (Taiyuan, China). Al powder was purchased from the Angang Group Aluminum Powder Co., Ltd. (Anshan, China). The nominal purity is 99%, with the impurities being mainly Si and Fe. The Al powder was screened through 300 mesh, 900 mesh and 2300 mesh screens, and particles of 20–54 μm and <5 μm were used. The chemical structures and physical-chemical properties of materials involved in the present work can be found in Table 1.

### 2.2. Preparation of FPU and FPU/Al/AP Propellant

**Scheme 1:**
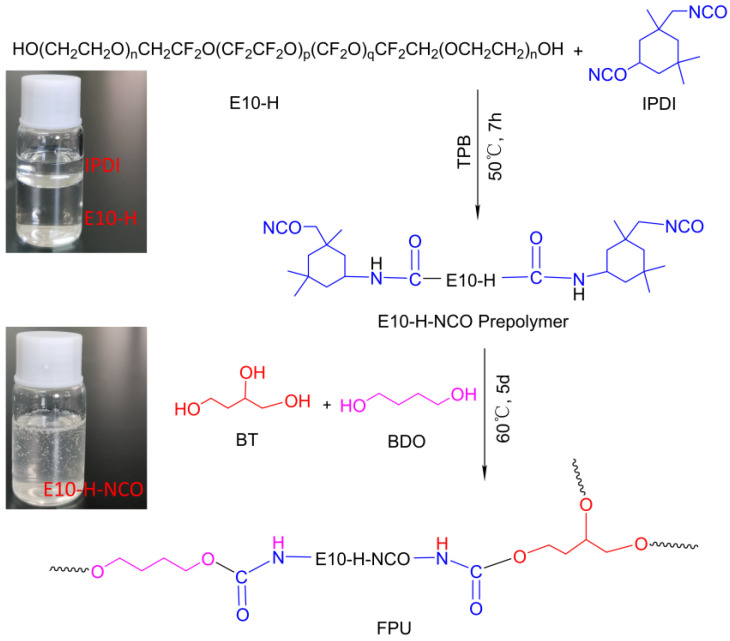
The synthesis procedure of fluorinated polyurethane (FPU).

Fluorinated polyurethane was prepared using E10-H as soft segments, IPDI as curing agent, BDO as chain extender and BT as crosslinker. The whole reaction process is depicted in Scheme 1. E-10H was first to react with IPDI to form the NCO-terminated prepolymer E10-H-NCO due to E10-H and IPDI being immiscible (as shown in the illustration in Scheme 1). Then, the prepolymer reacted with BDO and BT to complete the curing process. In this experiment, the reaction was performed in a three-necked flask equipped with a magnetic stirrer, an oil bath and a thermometer. E10-H (8 g), IPDI (2.22 g, 10 mmol) and TPB (5 mg, 0.05 wt%) were added to a three-necked flask and vigorously stirred at 50 °C for 7 h. Then, BT and BDO were added to the flask and stirred for 10 min to ensure uniform mixing. The molar ratio of OH/NCO in (E10-H+BT+BDO)/IPDI was fixed to 1/1. Finally, the liquid mixture was poured into a polytetrafluoroethylene mould and degassed for 15 min, followed by transfer in an oven at 60 °C for 5 days to complete the curing process. The formulations of FPU are shown in Table 2.

The propellant consisted of 14 wt% FPU binder, 70 wt% AP (80 wt% coarse 220 μm and 20 wt% fine 20 μm) and 16 wt% Al (20–54 μm). The propellant was mixed in a 20 g batch for 20 min using a vibratory mixer (ExLabSEM-I, Hubei Hangpeng Chemical Power Technology Co., Ltd., Hubei, China) at 60 G gravity. Then, the thick propellant slurry was directly cast into a quartz tube (dimensions: Φ 7 mm × 20 mm) and cured for about 5 days under 60 °C to obtain compact propellant samples. To study the effects of fluorine on the combustion properties of Al, HTPE-based polyurethane (HPU) was also prepared to compare the HPU/Al/AP propellant. HTPE is a kind of polyether prepolymer with a similar molecular skeleton (C-O-C) to E10-H [20]. HPU is one of the binders used in typical industrial propulsion [21]. The synthesis process of HPU was the same as that of FPU, replacing E10-H with HTPE only.

### 2.3. Characterization

FTIR spectroscopy was measured using a VERTEX70 spectrometer (Bruker, Werder Bremen, Germany), over a wavelength range of 4000–600 cm^−1^ at a resolution of 4.0 cm^−1^. The ^1^H NMR spectra of the specimens were measured on an AV-400 NMR spectrometer (Bruker, Werder Bremen, Germany) using deuterated dimethyl sulfoxide (DMSO-d6) as a solvent. The mechanical properties of FPU films were measured by an electronic universal testing machine (AGS-J, Shimadzu, Kyoto, Japan) at a constant strain rate of 100 mm/min at room temperature. The test samples were cut into dumbbell shapes of 20 mm (neck position length) × 4 mm (width) × 1 mm (thickness). Every sample was tested at least five times, and the average value was recorded. 

Thermal decomposition was analyzed by differential scanning calorimetry (DSC) thermogravimetry (TG), which was coupled with mass spectrometry (MS) analysis (STA449C and QMS 403C, Netzsch, Selb, Germany). Heating was performed from room temperature to 1200 °C at a heating rate of 10 K/min in Ar.

The FPU/Al/AP propellant was ignited using a CO_2_ laser under ambient conditions. The laser beam was directed to the specimen through a ZnSe lens with a focal length of 500 mm. The ignition combustion process was recorded using a high-speed video camera (i-SPEED 726, Essex, UK) at 1000 fps. Surface morphology of combustion products was observed using scanning electron microscopy (SEM, S4800, Hitachi, Tokyo, Japan), and elemental mapping measurements were obtained by energy-dispersive X-ray spectroscopy (EDS). The crystal structure was identified by powder X-ray diffraction (D8 FOCUS, Brucker, Werder Bremen, Germany) using nickel-filtered CuKα radiation (40 kV, 40 mA) at 2θ angles of 4–80°, scanning step = 0.02.

## 3. Results and Discussion 

### 3.1. Curing of FPU

**Figure 1:**
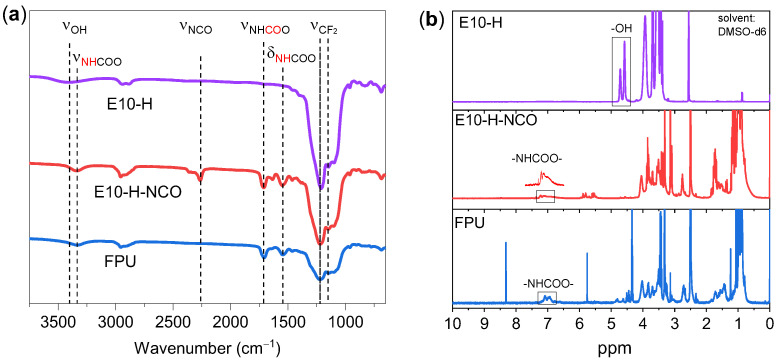
(**a**) FTIR spectra and (**b**) ^1^HNMR spectra of E10-H, E10-H-NCO and FPU-3.

FPUs with various -OH molar ratios of the chain extender (BDO) and crosslinker (BT) were prepared by a solvent-free two-step polymerization method. The chemical structures of E10-H, E10-H-NCO and FPU-3 were assessed by FTIR spectral analysis (Figure 1a). The peaks at 1145 cm^−1^ and 1211 cm^−1^ in the fingerprint spectrum correspond to CF_2_ symmetric stretching and CF_2_ asymmetric stretching [22]. The characteristic absorption peak at approximately 3420 cm^−1^ in E10-H corresponds to the -OH groups. After reaction with IPDI, four new peaks were observed in E10-H-NCO: 3329 cm^−1^ and 1546 cm^−1^ (stretching vibration and bending vibration of N-H in the -NHCOO- structure), 2264 cm^−1^ (stretching vibration of -NCO) and 1713 cm^−1^ (stretching vibration of C=O in the -NHCOO- structure) [23,24,25]. These results confirmed the successful preparation of the E10-H-NCO prepolymer. In the spectrum of FPU-3, the characteristic peak of the -NCO group at 2264 cm^−1^ disappears, which is a sign of its full conversion.

The occurrence of the polyurethane reaction was also evidenced by ^1^H NMR analysis. A comparison of the ^1^H NMR spectrum of FPU-3 with E10-H-NCO and E10-H is shown in Figure 1b. Regarding E10-H, the -OH groups give two peaks at 4.5–4.8 ppm [26]. The peaks at 6.9–7.1 ppm in both E10-H-NCO and FPU-3 represent the characteristic peaks of urethane NH groups [27]. The appearance of -NHCOO- peaks and the absence of -OH peaks indicate that the curing reaction of FPU occurred.

### 3.2. Mechanical Properties 

**Figure 2:**
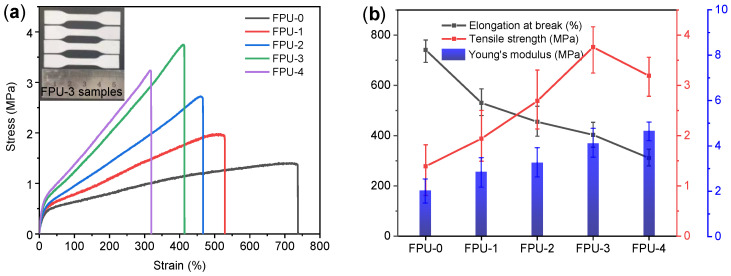
(**a**) Stress-strain curve of FPUs, and (**b**) tensile properties (tensile strength, elongation at break and Young’s modulus) of FPUs.

The mechanical properties are important and essential, they must be studied for the successful use of PU. Figure 2a displays the stress-strain curves of FPUs with various OH molar ratios of BDO/BT. FPU-0 showed the highest elongation at break (735%), and the minimum tensile strength (1.38 MPa). In contrast, FPU-3 showed the highest tensile strength of 3.7 MPa and a moderate elongation at break of 412%. The values of tensile strength and elongation at break obtained from the stress-strain curves of all FPUs are summarized along with the Young’s modulus data, as shown in Figure 2b. The Young’s modulus values were obtained from the slope of the linear portion of the stress-strain plot, where Hook’s law is obeyed. The elongation at break of the FPUs decreased with increasing BT content, whereas the tensile strength first increased and then decreased, and the Young’s modulus increased. This result shows the classical phenomenon of the trade-off of mechanical properties, which is primarily caused by the increase in BT content, which led to the increase in the chemical cross-linking network during the urethane curing reaction [28,29]. The interaction of crosslinking density resulted in an increase in tensile strength and Young’s modulus of the FPUs.

In the following work, FPU-3 was selected as the binder to be used in subsequent studies because it had the highest tensile strength and suitable elongation, which are necessary for binders of solid composite propellants and cast explosives.

### 3.3. Thermal Decomposition Behavior of FPU

**Figure 3:**
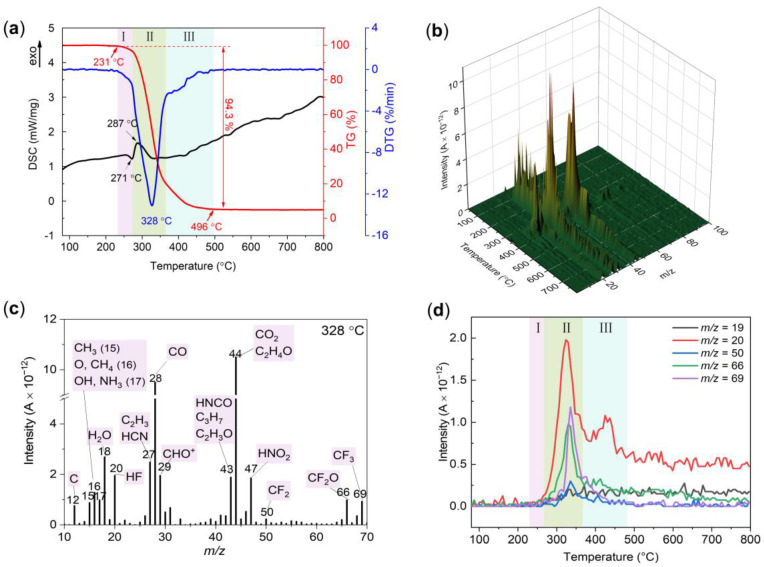
(**a**) DSC-TG-DTG curves of FPU in an argon atmosphere. (**b**) 3D pattern of ionic current intensity of decomposition gaseous products. (**c**) Ion current intensity of possible decomposition gaseous products at 328 °C. (**d**) The ion mass spectra of *m*/*z* = 19, 20, 50, 66 and 69.

The thermal decomposition behavior of the FPU was investigated by simultaneous DSC-TG-MS at a heating rate of 10 °C/min under an argon atmosphere. The DSC-TG-DTG curves of the FPU are shown in Figure 3a. The DSC curve only exhibited two weak peaks during the decomposition of PU, and an endothermic peak at approximately 271 °C succeeded by an exothermic peak at approximately 287 °C. Generally, all polyurethanes undergo a two-stage decomposition process, including the scission of hard segments and the cleavages of soft segments [30,31]. In this study, the decomposition of FPU is divided into three stages according to the weight loss of TG and DTG. The first stage from 231 to 271 °C corresponds to the depolymerization of the hard segments accompanied by an approximately 3.8% mass loss. The latter two stages correspond to the pyrolysis of the residual products. During the second stage, the residual products decompose rapidly and release gaseous products and other carbonaceous products. Remarkable FPU decomposition was observed by the sharp decrease in mass in the TG curve in the temperature range of 271–365 °C, accompanied by a maximum rate of mass loss at 328 °C in the DTG curve. Accordingly, several striking peaks of the ionic current intensity of decomposition gaseous products were observed at approximately 328 °C in the 3D pattern (Figure 3b).

The ion current intensity of the MS signals and their possible decomposition gaseous products at the DTG peak temperature is shown in Figure 3c. The main MS signals were found at *m*/*z* values of 44, 28, 18, 27, 29, 43, 47, 20, 66 and 69 in the order of descending intensity. As depicted in Figure 3c, *m*/*z* = 44 and 28 are the two main ion current peaks in the second stage. *m*/*z* = 28 can be attributed to CO, and *m*/*z* = 44 can be attributed to CO_2_ and C_2_H_4_O according to the molecular structure of FPU (Scheme 1). The large number of gaseous carbonaceous products detected by MS results indicates the rapid decomposition of the carbon skeleton at this temperature. Moreover, there are some fluorine fragments in gaseous products since *m*/*z* signals of 20 (HF), 50 (CF_2_), 66 (CF_2_O) and 69 (CF_3_) are detected. These fluorine fragments are strong oxidants and are favorable for the ignition and combustion of aluminum [16,32]. Figure 3d shows the ion current intensity of fluorine fragments vs. temperature curves. The onset and ending temperatures of the fluorine fragment intensity curves were almost the same as the temperature range of the second stage. Therefore, the decomposition of fluorine in FPU occurs mainly in the second stage. The third stage from 365 to 496 °C corresponds to the further degradation of the residual residue. Some HF (*m*/*z* = 20) is also released in the third stage (Figure 3d), which is beneficial for prolonging the reaction time between HF and aluminum.

### 3.4. Comparison of Combustion Properties of HPU/Al/AP and FPU/Al/AP Propellant

**Figure 4:**
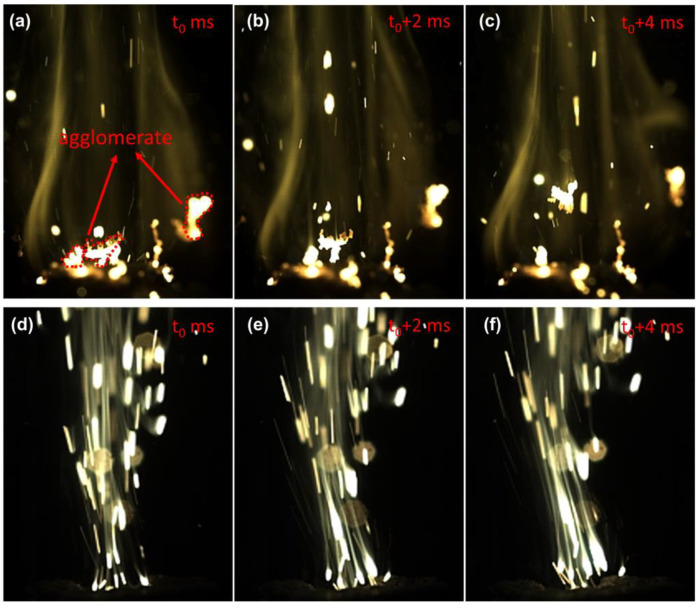
Images of the burning surfaces of different propellants at atmospheric pressure: (**a**–**c**) HPU/Al/AP; (**d**–**f**) FPU/Al/AP.

Figure 4 shows the high-speed video flame images during the laser ignition experiments of the FPU/Al/AP propellant and HPU/Al/AP propellant at atmospheric pressure. The time interval between the two selected successive frames was 2 ms. FPU has a dramatic effect on the combustion characteristics of the Al particles. Figure 4a–c shows the typical agglomeration processes of Al on the burning surface of the HPU/Al/AP propellant. The neighboring Al particles stick together and collapse into one fused drop, which is consistent with the experimental study of aluminum agglomeration of solid propellants reported by Wen et al. [5]. In contrast, no obvious agglomeration of the FPU/Al/AP propellant was observed. According to the analysis above, FPU decomposition will release fluorine fragments with strong oxidation. During FPU/Al/AP propellant heating, fluorine fragments and aluminum particles’ reaction can then produce AlF_3_ at the FPU/Al interfaces. AlF_3_ begins to sublime at 1276 °C, well below the combustion temperature of Al, which helps to reduce the agglomeration of molten Al particles [33].

**Figure 5:**
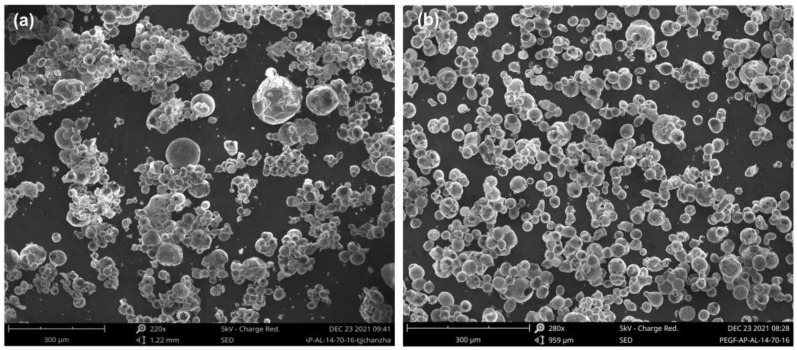
SEM images of condensed combustion products at atmospheric pressure: HPU/Al/AP (**a**); FPU/Al/AP (**b**).

The combustion products of the two aluminized propellants were collected in an open environment and observed by SEM (shown in Figure 5). As we expected, the FPU/AP/Al combustion products appeared to be in a similar size range to that of virgin Al, indicating that agglomeration did not occur appreciably. In contrast, HPU/AP/Al exhibited the highest agglomeration, reaching approximately 150 µm with a regular spherical shape. This result is consistent with previous experiments showing that Al particles on the burning surface of FPU/AP/Al propellants tend to be smaller than those on HPU/AP/Al propellants. 

**Figure 6:**
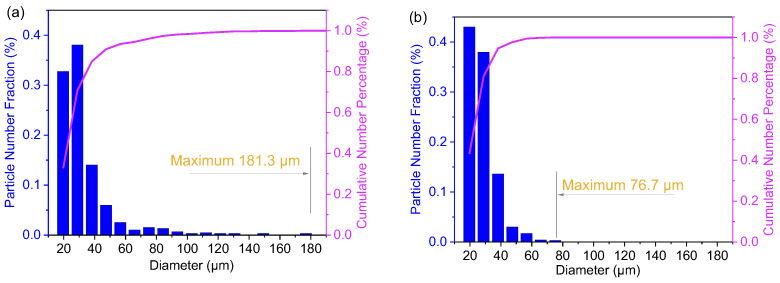
Size distribution of combustion products for the two propellants at atmospheric pressure: HPU/Al/AP (**a**); FPU/Al/AP (**b**).

The diameter of the combustion products was analyzed using Nano Measurer software. The size-distribution data of combustion products for the HPU/AP/Al propellant and FPU/AP/Al propellant are shown in Figure 6. The diameter of HPU/AP/Al is distributed in the range of 15–185 µm. A major part of the combustion products is below 50 µm, while the number of fractions of the agglomerates >60 µm is only 6.5%. It seems that there are few agglomerated Al particles, but when the agglomerate is regarded as a sphere, the agglomerate volume reaches about 61%. The agglomeration of Al particles leads to low propellant combustion efficiency and high two-phase flow losses. Compared with HPU/AP/Al, FPU/AP/Al results in a reduction in the maximum diameter size from 181.3 μm to 76.7 μm, which represents a decrease in the agglomerate volume of more than 10 times. This result suggests that the FPU binder can significantly reduce the agglomeration of Al particles in a solid propellant.

**Figure 7:**
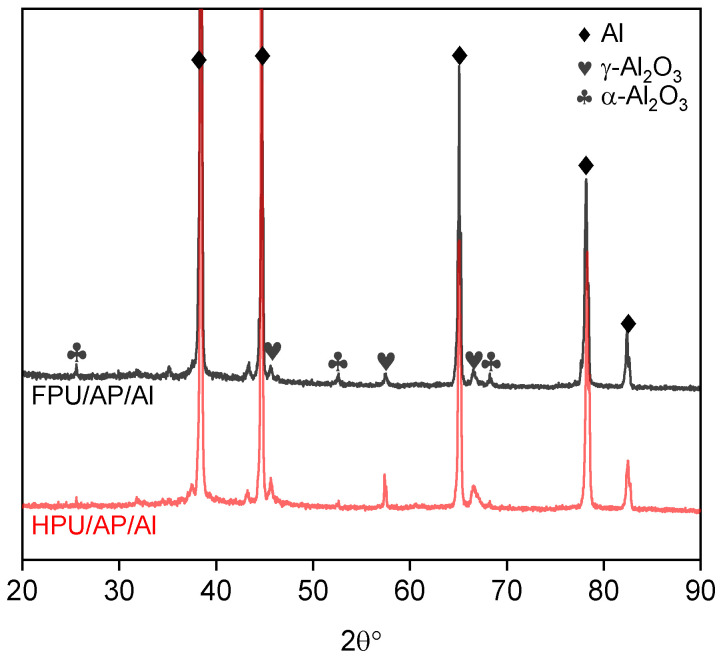
XRD patterns for combustion products for the two propellants.

The X-ray patterns of combustion products for the HPU/AP/Al propellant and FPU/AP/Al propellant are shown in Figure 7. Weak peaks of γ-Al_2_O_3_ and α-Al_2_O_3_, in addition to strong peaks of Al, are observed for both samples. The Al is the major phase due to the negative oxygen balance of propellants, resulting in the incomplete combustion of Al particles. Compared with HPU/AP/Al, the FPU/AP/Al propellant shows stronger α-Al_2_O_3_ peaks. This is due to the fact that the generated AlF_3_ promotes conversion of γ-Al_2_O_3_ to α-Al_2_O_3_, and the reaction mechanism is discussed later. However, the AlF_3_ peak was not observed in the XRD spectrum due to its consumption during the combustion process. 

### 3.5. Mechanism of Suppressing Agglomeration

**Figure 8:**
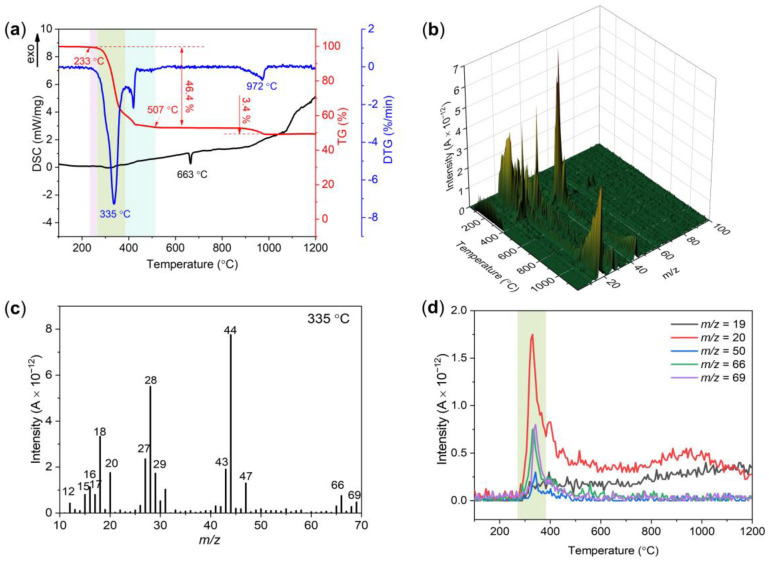
(**a**) DSC-TG-DTG curves of the FPU/Al under an argon atmosphere. (**b**) 3D pattern of ionic current intensity of decomposition gaseous products. (**c**) Ion current intensity of possible decomposition gaseous products at 335 °C. (**d**) The ion mass spectra of *m*/*z* = 19, 20, 50, 66 and 69.

To investigate the reaction mechanism between FPU and Al, the FPU/Al composite with mass ratio of 1:1 was prepared and tested by DSC-TG-MS method. The results are shown in Figure 8. The decomposition of FPU/Al is very similar to that of FPU in the temperature range of 100–800 °C. There is also no obvious endothermal or exothermic peak on the DSC curve, except for the melting peak of Al powder at 663 °C. The FPU decomposition process is also divided into three stages based on the weight loss of TG and DTG. According to the previous analysis of the thermal decomposition behavior of FPU, we know that fluorine fragments were mainly released in the second stage, as well as in the third stage for HF release. When Al is incorporated into FPU, the alumina shell of Al particles can react with fluorine fragments forms AlF_3_, which is referred to as the preignition reaction [32,34]. The fluorination of the Al_2_O_3_ shell exposes the Al core to allow for enhanced reaction kinetics. The following reactions outline main process in this temperature stage:

(1) $$\mathrm{FPU}\to C_{x}H_{y}O_{z}F_{m}{+\mathrm{HF}}_{\left(g\right)}$$

(2) $${\mathrm{Al}}_{2}O_{3}{+C}_{x}F_{y}O_{z}\to {\mathrm{AlF}}_{3(s)}{+\mathrm{CO}}_{2(g)}+\cdot \cdot \cdot$$

(3) $${\mathrm{Al}}_{2}O_{3}+\mathrm{HF}\to {\mathrm{AlF}}_{3(s)}+H_{2}O_{\left(s\right)}$$

As the temperature increased, an obvious mass loss peak was observed on the TG curve accompanied by a weight loss of 3.4% in the temperature range of 860–990 °C. In addition, the *m*/*z* = 20 curve shows a weak peak in the corresponding temperature range (Figure 8d), indicating the emission of HF in this temperature range. These results were similar to those attained by Riello et al. [35] in an AlF_3_ reaction mechanism study. The hydroxyl groups present in the transition alumina combine with AlF_3_ in the temperature range of 860–990 °C, by forming gaseous HF and AlOF, as shown in reaction (4). As such, HF could react with the transition alumina by generating the same intermediate compound AlOF as shown in reaction (5). Finally, the gaseous AlOF species nucleate and grow α-Al_2_O_3_ at specific nucleation sites, releasing fluorine, as shown in reaction (6).

(4) $${\mathrm{AlF}}_{3(s)}+3H_{2}O_{\left(v\right)}\to {\mathrm{AlOF}}_{\left(g\right)}+2{\mathrm{HF}}_{\left(g\right)}$$

(5) $$2{\mathrm{HF}}_{\left(g\right)}+{\mathrm{Al}}_{2}O_{3(s)}\to 2{\mathrm{AlOF}}_{\left(g\right)}+H_{2}O_{\left(v\right)}$$

(6) $$2{\mathrm{AlOF}}_{\left(g\right)}+H_{2}O_{\left(v\right)}\to \alpha {-\mathrm{Al}}_{2}O_{3(s)}+2{\mathrm{HF}}_{\left(g\right)}$$

**Figure 9:**
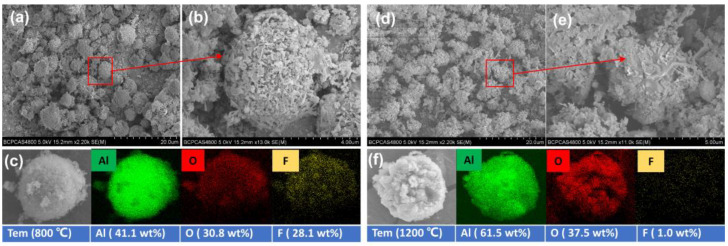
Decomposition residue at 800 °C for low- and high-resolution SEM (**a**,**b**), and EDS mapping (**c**). Decomposition residue at 1200 °C for low- and high-resolution SEM (**d**,**e**), and EDS mapping (**f**).

Figure 9 shows the SEM and EDS images of the decomposition residue at 800 °C and 1200 °C in under an argon atmosphere. The decomposed residue is spherical for approximately 5 µm, maintaining the structure of raw Al. Element mapping results reveal that the decomposition product at 800 °C has more F elements than that at 1200 °C, which is consistent with the previous DSC-TG-MS analysis.

**Figure 10:**
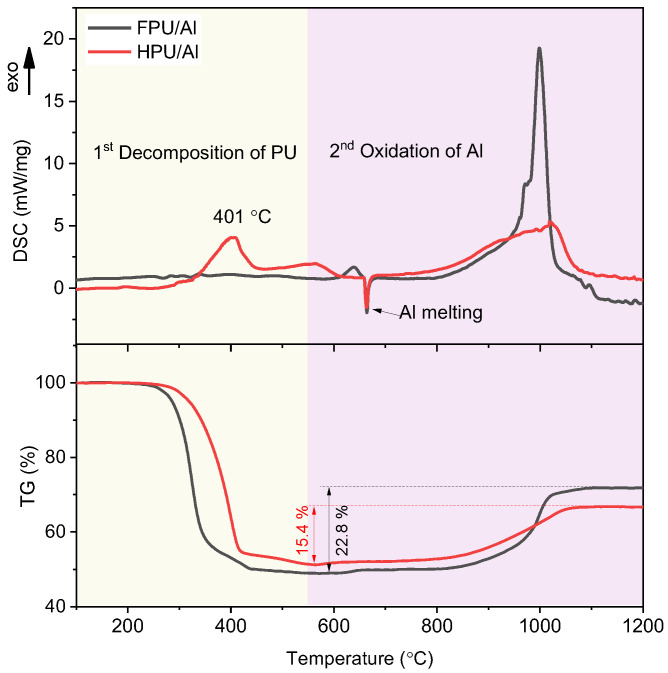
DSC-TG curves of FPU/Al and HPU/Al in an air atmosphere.

To carry out a more in-depth analysis of Al oxidation by a gas phase product from the decomposition of FPU, HPU/Al was prepared and compared with FPU/Al. The decomposition of FPU/Al and HPU/Al was tested by TG-DSC with a heating rate of 10 °C/min under an air atmosphere. As shown in Figure 10, the overall reaction processes of FPU/Al and HPU/Al were completed in two stages in the temperature range of 100 °C–1200 °C. The first stage in the temperature range of 100–550 °C corresponds to the decomposition of PU. The DSC curve of FPU/Al in an air atmosphere is similar to that in an argon atmosphere without any obvious change in enthalpy. Additionally, the mass loss of FPU/Al in this temperature range is approximately 51 wt%, which is consistent with the composition of the raw material. The first stage in the temperature range of 550–1200 °C corresponds to the weight gain of Al during the oxidation process. Unlike the decomposition under inert conditions, FPU/Al shows a steep exothermic peak in the DSC curve in the temperature range of 780–1035 °C due to the oxidation of Al. In contrast, the exothermic peak of HPU/Al in this temperature range is milder. This result indicates that the Al in FPU/Al is more active than that in HPU/Al since the strong oxidizing HF released by FPU during the first stage can react with the alumina shell of Al. In addition, the weight gain of FPU/Al is 22.1% and that of HPU/Al is 14.6%, which can also be inferred to indicate that the reaction of FPU/Al is more complete.

**Figure 11:**
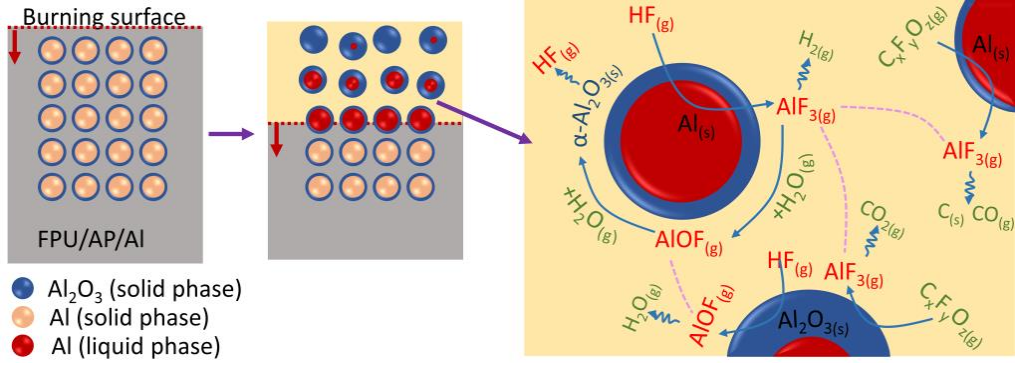
Schematic of the corresponding mechanism of FPU suppressing agglomerate.

Figure 11 illustrates a schematic of the corresponding mechanism of a typical suppressing agglomerate. From the DSC-TG-MS analysis, it was possible to detect the emission of HF, CF_2_, CF_3_, CF_2_O, etc., vapor during the FPU decomposition. These high-active gases react with Al directly to form AlF_3_. The AlF_3_ is in gaseous state during combustion due to its low boiling point, which is conducive to preventing the accumulation of Al particles. On the other hand, the generated AlF_3_ is an excellent catalyst in the gas-solid reaction of transition alumina to the alpha alumina phase [35]. In this process, HF reacts with Al to form AlF_3_, and AlF_3_ reacts with H_2_O to form AlOF, which is finally converted into α-Al_2_O_3_ and releases HF. HF will participate in the next round of reaction. These gaseous products are constantly transformed on the burning surface of Al and prevent the sticking of Al particles.

## 4. Conclusions

In this experimental study, FPU was prepared successfully for use as a potential binder to reduce the agglomeration of Al particles in aluminized propellants. Of particular interest are the mechanical, thermal decomposition and oxidation characteristics of FPU and its effect on aluminized propellant agglomeration. Mechanical tests showed that the tensile strength of the optimal FPU is 3.7 MPa and the breaking elongation is 412%. Thermal decomposition analysis results showed that strong oxidizing fluorine fragments such as HF, CF_2_, CF_3_ and CF_2_O are released during FPU decomposition. These oxidizing gases react with Al to form AlF_3_. More importantly, the application of FPU binder to aluminized propellant was found to efficiently reduce the agglomeration of Al particles. Since the AlF_3_ is easy to vaporize and the AlF_3_ acts by a gas-phase mechanism of transition alumina to the alpha alumina phase. This work provides a new viewpoint regarding the fabrication of FPU binders and their potential application in aluminized propellants.

## Figures and Tables

**Table 1:** Properties of involved materials.

Samples	Molecular Formula	Molecular Weight	Density (g/cm^3^)	Roles
E10-H	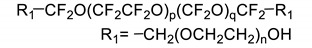	750	1.73	soft segment
HTPE	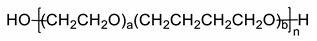	2800	1.04	soft segment
IPDI	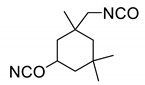	222.3	1.06	curing agent
BDO	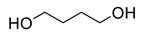	90.1	1.02	chain extender
BT	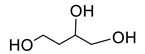	106.1	1.18	crosslinker
AP	NH_4_ClO_4_	117.5	1.95	oxidizer
Al		27	2.7	fuel

**Table 2:** Sample code designation and formulation of FPU.

Samples	E10-H/g	IPDI/g	-OH Ratio of BDO/BT	BDO/g	BT/g
FPU-0	8	2.22	4/0	0.54	0
FPU-1	8	2.22	3/1	0.405	0.106
FPU-2	8	2.22	2/2	0.27	0.212
FPU-3	8	2.22	1/3	0.135	0.318
FPU-4	8	2.22	0/4	0	0.424

## Data Availability

The data presented in this study are available on request from the corresponding author.

## Abbreviations

FPU	fluorinated polyurethane
IPDI	isophorone diisocyanate
BT	1,2,4-butanetriol
BDO	1,4-butanediol
HTPE	hydroxyl-terminated polyether
HPU	HTPE-based polyurethane
E10-H	dialcohol terminated perfluoropolyether
E10-H-NCO	NCO-terminated E10-H
TPB	triphenylbismuth
AP	ammonium perchlorate
